# Co-Occurrence of Sarcopenia and Frailty in Acutely Admitted Older Medical Patients: Results from the Copenhagen PROTECT Study

**DOI:** 10.14283/jfa.2024.23

**Published:** 2024-03-19

**Authors:** Hanne Nygaard, R.S. Kamper, A. Ekmann, S.K. Hansen, P. Hansen, M. Schultz, J. Rasmussen, E. Pressel, C. Suetta

**Affiliations:** 1 Department of Emergency Medicine, Copenhagen University Hospital, Bispebjerg and Frederiksberg, Copenhagen, Denmark; 2 CopenAge, Copenhagen Center for Clinical Age Research, University of Copenhagen, Copenhagen, Denmark; 3 Department of Geriatric & Palliative Medicine, Copenhagen University Hospital, Bispebjerg and Frederiksberg, Copenhagen, Denmark; 4 Department of Medicine, Copenhagen University Hospital, Herlev and Gentofte, Herlev, Denmark; 5 Ebba Lunds Vej 40A, 2400, Copenhagen, Denmark

**Keywords:** Ageing, physical function, mortality, acute, geriatric

## Abstract

**Background:**

Sarcopenia and frailty are often used interchangeably in clinical practice yet represent two distinct conditions and require different therapeutic approaches. The literature regarding the co-occurrence of both conditions in older patients is scarce as most studies have investigated the prevalence of sarcopenia and frailty separately.

**Objectives:**

We aim to evaluate the prevalence and co-occurrence of sarcopenia and frailty in a large sample of acutely admitted older medical patients.

**Design:**

Secondary analyses using cross-sectional data from the Copenhagen PROTECT study.

**Setting:**

Patients were included from the acute medical ward at Copenhagen University Hospital, Bispebjerg and Frederiksberg, Copenhagen, Denmark, between November 2019 and November 2021.

**Participants:**

Acutely admitted older medical patients (≥65 years).

**Measurements:**

Handgrip strength (HGS) was investigated using a handheld dynamometer. Lean mass (SMI) was investigated using direct-segmental multifrequency bioelectrical impedance analyses (DSM-BIA). Low HGS, low SMI, and sarcopenia were defined according to the recent definitions from the European Working Group on Sarcopenia in Older People (EWGSOP2). The Clinical Frailty Scale (CFS) was used to evaluate frailty, with a value > 5 indicating the presence of frailty. Patients were enrolled and tested within 24 hours of admission.

**Results:**

This study included 638 patients (mean age: 78.2±7.6, 55% female) with complete records of SMI, HGS, and the CFS. The prevalence of low HGS, low SMI, sarcopenia, and frailty were 39.0%, 33.1%, 19.7%, and 39.0%, respectively. Sarcopenia and frailty co-occurred in 12.1% of the patients.

**Conclusions:**

It is well-known that sarcopenia and frailty represent clinical manifestations of ageing and overlap in terms of the impairment in physical function observed in both conditions. Our results demonstrate that sarcopenia and frailty do not necessarily co-occur within the older acutely admitted patient, highlighting the need for separate assessments of frailty and sarcopenia to ensure the accurate characterization of the health status of older patients.

## Background

**S**arcopenia and frailty are common conditions in the older population (1, 2). Sarcopenia is the condition of age-related loss of muscle mass and strength, playing a major role in the functional impairment that may occur with old age (3, 4, 5, 6, 7, 8, 9, 10). Frailty, on the other hand, is a multidimensional clinical condition characterized by a decrease in biological reserve capacity leading to increased vulnerability and reduced ability to resist stressors such as illness, falls, or any circumstances that affect mental and physical well-being (11, 12, 13, 14). Both frailty and sarcopenia are associated with adverse outcomes such as functional impairments, recurrent falls, hospitalization, and mortality (11, 12, 15, 16, 17). Furthermore, both conditions share etiological factors such as malnutrition, inflammation, hormonal changes, and reduced physical activity (18). The physical function impairment has been described as a possible core element shared by the two conditions (19), which can co-occur within the same individual (20).

Following the initial research in frailty and sarcopenia approximately 20 years ago, these conditions have been investigated in parallel (21). Although sarcopenia and frailty are often used interchangeably in clinical practice, they represent two distinct conditions and require different therapeutic approaches. Furthermore, the prevalence and co-occurrence of the two conditions depend on the used definitions (20, 22, 23). The co-occurrence of sarcopenia and frailty have previously been investigated in community-dwelling older adults (22, 23, 24, 25, 26). However, the literature regarding the co-occurrence in older in-hospital patients is scarce as most studies have investigated the prevalence of sarcopenia and frailty separately (27).

A previous study regarding the prevalence and overlap of sarcopenia and frailty in a small cohort of older patients demonstrated that sarcopenia and frailty co-occurred in more than half of the patients (10, 20). However, the criteria for defining sarcopenia were different from the currently accepted definition from the European Working Group on Sarcopenia in Older People (EWGSOP2) (10), and the study assessed frailty using the definition by Fried et al (28). Notably, more comprehensive measures such as gait speed included in the definition by Fried may not be suitable for all acutely admitted medical patients who may be immobile or affected by their acute illness (29). Indeed, a recent study evaluating the utility of several frailty screening tools concluded that the Fried definition showed the lowest feasibility in the clinical setting, and that the Clinical Frailty Scale (CFS) among others is a suitable choice for evaluating frailty in the clinical setting (30).

As such, we aim to evaluate the prevalence and co-occurrence of sarcopenia and frailty in a large sample of acutely admitted older medical patients using the recent guidelines from EWGSOP2 (10) and the CFS by Rockwood (31). Additionally, we aim to characterize the groups of patients classified as ‘Neither frail nor sarcopenic’, ‘Frail only’, ‘Sarcopenic only’, and ‘Both frail and sarcopenic’, and to investigate the predictive ability of the presence of these conditions with 90-day all-cause mortality post admission.

## Methods and materials

### Study design and participants

The current study used baseline data from the Copenhagen PROTECT study, a prospective cohort study including acutely admitted older (≥65 years) medical patients at Copenhagen University Hospital, Bispebjerg and Frederiksberg (BFH), between November 2019 and November 2021. The healthcare system in Denmark is fully tax-funded where in-hospital diagnostics and treatments are carried out without direct costs to the patient (32) BFH is an acute intake community hospital in the capital of Denmark, Copenhagen with a catchment area of about 483.000 inhabitants (33). The acute medical ward covers the following medical specialties: lung- and infectious medicine, endocrinology, geriatric medicine, gastroenterology, and acute medicine (34). Patients were recruited from the acute medical ward and data were collected within 24 hours of admission. A detailed description of the study protocol can be viewed elsewhere (35).

This study included a subset of patients with a full data set on both sarcopenia measures and frailty (n=638). Patient characteristics included age, sex, body mass index (BMI), number of prior hospitalizations within the last year until index admission, residential dependency (own home/nursing home) prior to admission, and discharge destination (own home / nursing home / rehabilitation stay). Comorbidity was evaluated by the Charlson Comorbidity Index (CCI) (36), and polypharmacy was present when the number of prescribed medications >5 (35). Risk of malnutrition was evaluated in a subsample (n=412) using the Short Nutritional Assessment Questionnaire (SNAQ) (37). Fall incidents within the last year until index admission were reported by the patient and classified as either no falls, 1–3 falls, or 4 or more falls.

### Lean mass, muscle strength, sarcopenia & frailty

As proposed by the EWGSOP2, patients were screened for potential sarcopenia using the Strength, Assistance with walking, Rising from a chair, Climbing stairs, and Falls questionnaire (SARC-F) (38). Potential sarcopenia was present at SARC-F scores ≥ 4. Regardless of the SARC-F score, the following measurements were performed. Appendicular lean mass (ALM) was assessed using direct-segmental multifrequency bioelectrical impedance analyses (DSM-BIA) (Inbody S10, BioSpace, Ltd., Seoul, South Korea) and relative ALM (kg/height^2^) was reported as the skeletal muscle index (SMI). Low lean mass was defined as an SMI less than 5.5 kg/m2 and 7.0 kg/m2 for women and men, respectively, using the cut-offs from the EWGSOP2 (10). Handgrip strength (HGS) was assessed using a digital hand-held dynamometer (Model SH1001; SAEHAN Corporation, Yangdeok-Dong, Masan, South Korea) as the highest value of three attempts with the dominant hand (35). Low HGS was defined as a HGS less than 27 kg and 16 kg for men and women, respectively, using the cut-offs from the EWGSOP2 (10). Sarcopenia was reported as a dichotomous variable as the presence of low handgrip strength with concurrent low SMI. Frailty was assessed using the 9-point CFS, according to the definition by Rockwood et al (31). A frailty score ≥ 5 indicated the presence of frailty.

### Outcome of interest

The primary outcome of interest was 90-day all-cause mortality following admission. Information regarding mortality was obtained from the Danish Civil Registration System (39).

### Statistics

Descriptive statistics were performed to determine prevalence rates of low SMI, low HGS, sarcopenia, and frailty and reported as the relative frequency (%). Patients were stratified into four groups according to the presences of frailty and sarcopenia i.e., ‘Neither frail nor sarcopenic’, ‘Frail only’, ‘Sarcopenic only’, and ‘Both frail and sarcopenic’. The group of patients with neither frailty nor sarcopenia was used as reference when comparing differences between the groups. Patient characteristics were reported as mean ± SD or relative frequency (%). Differences in patient characteristics were assessed using independent t-tests, or Chi2 with subsequent post-hoc Bonferroni corrected z-tests. Cox regression analysis was used to model survival in the four frail/sarcopenic groups and was reported as hazard ratio (HR) with 95% confidence intervals (95% CI). Person-days of follow-up were calculated from date of hospital admission to date of death or end at follow-up (90 days). The model was reported both as unadjusted models, a model adjusted for age and sex and model adjusted for age, sex, and the CCI. Proportional hazard assumptions were statistically tested and found satisfactory.

Data were analyzed using IBM SPSS Statistics (version 29.0). The level of significance was set at p<0.05.

## Results

A total of 638 patients with complete records of both HGS, SMI, and the CFS were included in the study (mean age 78.2±7.6, 55% female). The prevalences are presented in Table 1.

**Table 1:** Prevalence of low HGS, low SMI, sarcopenia, & frailty

	All patients
Low HGS, n (%)	249 (39.0%)
Low SMI, n (%)	211 (33.1%)
Sarcopenia, n (%)	126 (19.7%)
Frailty, n (%)	249 (39.0%)


Abbreviations: HGS (Handgrip strength), SMI (skeletal muscle index)

Patient characteristics of the total sample and according to the presence of frailty and sarcopenia are presented in Table 2. Compared to the group of patients that were neither frail nor sarcopenic, patients with frailty and/or sarcopenia were significantly older, had a significantly lower HGS, a significantly higher proportion of patients at risk of severe malnutrition, and a significantly larger proportion of patients with potential sarcopenia evaluated by SARC-F. Groups including sarcopenic patients, i.e., the ‘Both frail and sarcopenic’ group and ‘Sarcopenic only’, had a significantly lower BMI and a lower SMI compared to the reference group. The prevalence of severe comorbidity and polypharmacy were significantly higher in groups with frail patients i.e. the ‘Both frail and sarcopenic’ and the ‘Frail only’ compared to the reference group. Furthermore, groups with frail patients also demonstrated a higher prevalence of four or more self-reported falls within the last year and a higher prevalence of two or more hospitalizations during the year up until the index admission. All patients discharged to nursing homes were classified as frail. Likewise, groups with frail patients had significantly less patients who were discharged to their own home.

**Table 2:** Patient characteristics of the total sample and according to the presence of frailty and sarcopenia

		Total sample (n=638)	Neither frail nor sarcopenic (n=340)	Frail only (n=172)	Sarcopenic only (n=49)	Both frail and sarcopenic (n=77)
Age (years)		78.2±7.6	76.4±6.8	79.5±7.6*	79.4±8.7*	82.1±8.0*
Female sex (%)		55.2%	53%	62.2%	40.8%	57.1%
BMI (kg/m^2^)		25.6±5.2	26.7±5.0	26.3±5.3	22.5±3.3*	21.5±3.5*
Polypharmacy (%)		70.8%	64.1%	83.7%*	57.1%	80.5%*
Potential Sarcopenia (SARC-F) ‡ (%)		45.1%	21.2%	75.0%*	52.5%*	87.5%*
HGS (kg)						
	Female	18.0±6.0	20.7±5.6	17.2±4.9*	13.2±1.9*	11.3±3.4*
	Male	28.7±8.6	32.7±7.9	27.2±6.5*	21.6±4.3*	19.1±5.3*
SMI (kg/m^2^)						
	Female	6.1±1.2	6.4±1.1	6.4±1.2	4.8±0.5*	4.7±0.6*
	Male	7.6±1.3	8.0±1.1	8.0±1.3	6.3±0.5*	6.1±0.6*
Risk of malnutrition† (%)						
	Moderately malnourished	7.8%	6.4%	8.9%	16.1%	5.9%
	Severely malnourished	26.2%	17.0%	34.8%*	32.3%*	43.1%*
Comorbidity (%)						
	Mild	3.9%	5.3%	1.7%	8.2%	0.0%*
	Moderate	42.3%	50.9%	33.7%*	40.8%	24.7%*
	Severe	53.8%	43.8%	64.5%*	51.0%	75.3%*
Fall incidents‡ (%)						
	No falls	49.7%	62.6%	28.9%*	50.0%	33.9%*
	1–3 falls	35.0%	31.9%	39.8%	40.0%	35.7%
	4 or more	15.3%	5.5%	31.3%*	10.0%	30.4%*
Prior hospitalizations (%)						
	0	52.0%	62.1%	38.8%*	56.3%	33.8%*
	1	25.6%	23.4%	27.1%	22.9%	33.8%
	≥2	22.4%	14.5%	34.1%*	20.8%	32.4%*
Residential dependency (%)						
	Own home	95.6%	100%	92.5.8%*	100%	80.5%*
	Nursing home	4.4%	0.0%	7.6%*	0.0%	19.5%*
Discharge destination§ (%)						
	Own home	86.5%	95.8%	73.8%*	91.8%	69.3%*
	Rehabilitation	8.3%	4.2%	16.7%*	8.2%	8.0%
	Nursing home	5.3%	0.0%	9.5%*	0.0%	22.7%*


Values are expressed as mean±SD or as percentages. *Significantly different from the “Neither frail nor sarcopenic” group at p<0.05. †n=412 of total sample, ‡n=497 of total sample, §n=628 of total sample. Risk of malnutrition: score of 2 (moderately malnourished), score ≥3 (severely malnourished). Comorbidity: score of 1–2 (mild comorbidity), score of 3–4 (moderate comorbidity), score of ≥ 5 (severe comorbidity). Polypharmacy: present if number of prescribed medications > 5. Potential Sarcopenia (SARC-F): present at scores ≥ 4. Abbreviations: BMI (Body mass index), F (frailty), HGS (Handgrip strength), S (sarcopenia), SARC-F (Strength, Assistance with walking, Rising from a chair, Climbing stairs, and Falls questionnaire), SMI (skeletal muscle index)

**Figure 1:**
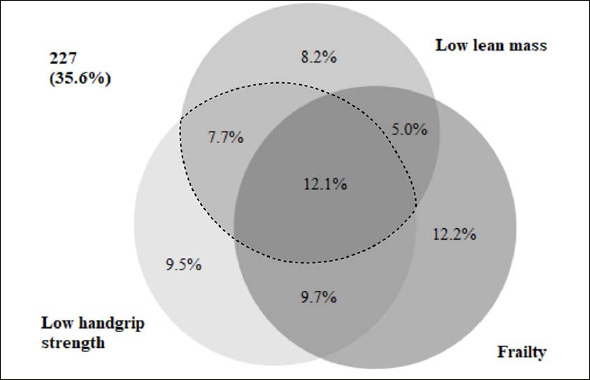
Proportional Venn-diagram displaying the co-occurrence of frailty, low handgrip strength, and low lean mass in acutely admitted older patients

The co-occurrence of low HGS, low SMI, and frailty is illustrated in a proportional Venn diagram (40) (Figure 1). Of the 249 patients with frailty, 56% had concurrent low HGS, 44% had concurrent low SMI, and 31% had concurrent sarcopenia. Frailty co-occurred in 61% of the 126 patients with sarcopenia. Of the 638 patients, 35.6% had neither low HGS, low SMI, nor frailty. The co-occurrence of sarcopenia and frailty was evident in 12.1% of the total population.

The dashed line frames the group of patients with sarcopenia.

### Risk of mortality 90 days post admission

The incident proportion of 90-day post admission mortality was 6.8%, 13.4%, 12.2%, and 15.6% for the ‘Neither frail nor sarcopenic’, ‘Frail only’, ‘Sarcopenic only’, and ‘Both frail and sarcopenic’ groups, respectively. Compared to the reference group, the ‘Frail only’ and ‘Both frail and sarcopenic’ groups demonstrated a significantly higher risk of all-cause mortality 90-day post admission (both p<0.05) (Table 3). In a second model adjusted for the effect of age and sex, the higher risk of mortality remained statistically significant in the ‘Frail only’ (p<0.05), while the higher risk of mortality in the ‘Both frail and sarcopenic’ group was not statistically significant (p=0.06). Compared to the reference group, the ‘Frail only’, ‘Sarcopenic only’, and the ‘Both frail and sarcopenic’ groups did not demonstrate an increased risk of all-cause mortality 90-day post admission in a third model adjusted for age, sex, and comorbidity (Table 3).

**Table 3:** Relationship of the prevalence and co-occurrence of frailty and sarcopenia with survival 90-day post admission

	Total n (%)	Died n (%)	Model 1 HR (95% CI)	p-value	Model 2 HR (95% CI)	p-value	Model 3 HR (95% CI)	p-value
Neither frail nor sarcopenic	340 (53.3)	23 (6.8)	1		1		1	
Frail only	172 (27.0)	23 (13.4)	1.97 (1.10, 3.54)	0.02*	1.91 (1.06, 3.47)	0.03*	1.37 (0.75, 2.50)	0.30
Sarcopenic only	49 (7.7)	6 (12.2)	1.87 (0.76, 4.60)	0.17	1.47 (0.59, 3.64)	0.41	1.36 (0.54, 3.42)	0.51
Both frail and sarcopenic	77 (12.1)	12 (15.6)	2.43 (1.21, 4.88)	0.01*	2.00 (0.97, 4.13)	0.06	1.35 (0.65, 2.82)	0.42


Analyses are performed using Cox regression in an unadjusted model (model 1), a model adjusted for age and sex (model 2) and a model adjusted for age, sex, and comorbidity (model 3). *Significantly different from the “Neither frail nor sarcopenic” group at p<0.05. Abbreviations: CI (Confidence Interval); HR (Hazard Ratio)

## Discussion

The pathophysiological similarities between sarcopenia and frailty, and the physical function impairment, which has been described as a possible core element shared by the two conditions, has led to a continuous debate regarding the causal relationship between the two (19). Perhaps the point is not determining whether one is due to the other, but rather defining the aspects and characteristics shared by patients with or without the conditions. In the present study, we aimed to evaluate the prevalence and co-occurrence of sarcopenia and frailty in a large sample of acutely admitted older medical patients using the recent guidelines from EWGSOP2 (10) and The Clinical Frailty Scale by Rockwood (31). Frailty was a common condition present in more than 1/3 of older patients. Sarcopenia had a prevalence of approximately 20%, whereas the elements of sarcopenia, low HGS and low SMI, were present in 39% and 30% of older patients, respectively. This is in line with another study on geriatric outpatients demonstrating that the prevalence of frailty was higher than the prevalence of sarcopenia (22).

### Co-occurrence of frailty and sarcopenia

Previous research has demonstrated a need for separate assessments of both frailty and sarcopenia to ensure the accurate characterization of the health status of older people (23, 41). While the majority of these studies have been performed on community-dwelling older people, the literature regarding the co-occurrence of both conditions in hospitalized older patients is scarce as most studies have investigated the prevalence of sarcopenia and frailty separately (27). Notably, the characterization of patients presenting with frailty and/or sarcopenia may aid in risk assessment in clinical practice.

Although frailty and sarcopenia are common conditions, they do not necessarily co-occur within the older acutely admitted patient (Figure 1). In fact, our data, and the study by Reijnierse et al, show that sarcopenic patients are more likely to be frail than frail patients to be sarcopenic (22). In contrast, other studies have found frail older people more likely to be sarcopenic than sarcopenic older people to be frail (24, 42, 43), which may be caused by differences in definitions, assessments, cut-off values, and study populations. Indeed, some studies have demonstrated a higher co-occurrence of sarcopenia and frailty assessed using the Fried definition (22, 28). This is expected as the Fried definition views frailty as physical frailty as opposed to Rockwood who defines frailty as a “cumulation of deficits” indicating that patients are at greater risk when they have several health deficits (12, 31). From this perspective, frailty can be viewed as an accumulation of risk factors for adverse events ultimately leading to greater risk of hospitalizations, increased use of health care services, and mortality (44). We have previously demonstrated a low concordance between frailty assessed by the Fried definition and the Clinical Frailty Scale (CFS), and a better prognostic ability of the CFS in terms of predicting 90-day mortality in older acutely admitted patients (45, 46). In this regard, the Clinical Frailty Scale is receiving increased attention as a screening tool in clinical practice and is often used in the hospital setting due to the feasibility and ease-of-use (30, 47). Notably, the low concordance between frailty screening tools, as well as differences in prevalence rates and predictive abilities could indicate the presence of several subtypes of frailty (30).

Our data demonstrate that patients with neither frailty nor sarcopenia had a significantly higher mean handgrip strength and a significantly lower prevalence of severe malnutrition compared to patients with a condition, regardless of whether they were frail only, sarcopenic only, or both frail and sarcopenic. This supports the notion that physical function impairment is a shared feature of both conditions (19) yet emphasize the need for the separate assessment of frailty and sarcopenia as they are both associated with adverse outcomes. To our surprise, the prevalence of potential sarcopenia, evaluated by SARC-F, was higher in groups including frail patients (i.e., the group of frail only and the group with both frail and sarcopenic patients) compared with sarcopenic only patients. Whether the SARC-F screening has better discriminative performance for frailty than sarcopenia should be investigated in a future study.

The prevalence of severe comorbidity and polypharmacy were significantly higher in groups with frail patients i.e. the group of frail only patients and the group of both frail and sarcopenic patients compared to the reference group. This was also reflected as a higher proportion of frail patients with two or more hospitalizations during the year up until the index admission compared to the reference group. These results highlight the increased health deficits of frail patients and underlines the need for the systemic assessment of frailty for the identification of patients at risk.

### Mortality risk

The incident proportion of all-cause mortality was approximately twice as high in groups of patients displaying either frailty, sarcopenia, or a combination of the two. Notably, as demonstrated by the results from the Cox regression analyses, only groups entailing patients with frailty (i.e. the ‘Frail only’ and ‘Both frail and sarcopenic’ groups) exhibited a higher risk of all-cause 90-day post admission mortality compared to patients with neither frailty nor sarcopenia. Being classified as frail appeared to entail a two-fold increased risk of dying following an acute admission, with hazard ratios of 2.4 and 2.0 for the ‘Frail only’ and ‘Both frail and sarcopenic’ groups, respectively (both p<0.05). However, in analyses adjusted for the effect of age and sex, only the ‘Frail only’ group remained statistically significant (HR 1.91, p<0.05), potentially reflecting a type II error caused by insufficient power in the ‘Both frail and sarcopenic group’ (HR 2.00, p=0.06).

In a third model adjusted for the effect of age, sex, and comorbidity, neither group displayed a significantly increased risk of all-cause mortality following 90-day of admission. Thus, when comorbidity is taken into account, it appears that frailty and sarcopenia does not add additional prognostic value in terms of predicting 90-day post admission all-cause mortality risk in the acute setting. Conversely, the study by Thompson et al found that community-dwelling older individuals with both frailty and sarcopenia demonstrated a significantly higher risk of 10-year mortality compared to older individuals with neither frailty nor sarcopenia in multivariable analyses adjusted for the effect of age, sex, and multimorbidity among others (26). The discrepancy in results may reflect differences in follow-up time, population characteristics, or an effect of acute admission. Indeed, the need for acute health care service significantly increases the risk of 1-year mortality (48).

### Limitations

This study has some limitations that needs to be addressed. One must take into account that this study is based on patients with complete records of both frailty, handgrip strength, and body composition. As the main reason for missing measurements entailed an inability to mobilize patients from their hospital bed to obtain the body weight needed for the body composition algorithm (49), it is possible that the prevalence of frailty and sarcopenia, and thus the co-occurrence of the conditions, is even higher than reported here. Of note, the prevalence of frailty in the total cohort was 48%, with low HGS co-occurring in 63% of these patients (data not shown). Here we report that frailty was present in 39% of the patients, with low HGS co-occurring in 56% of these patients. Although the prevalence of frailty and sarcopenia are potentially higher in the total cohort, the co-occurrence of both conditions appear fairly similar.

## Conclusion

It is well-known that sarcopenia and frailty represent clinical manifestations of ageing and overlap in terms of the impairment in physical function observed in both conditions. Notably, when comorbidity is taken into account, frailty and sarcopenia does not add additional prognostic value in terms of predicting 90-day post admission all-cause mortality risk in the acute setting. Our results demonstrate that sarcopenia and frailty do not necessarily co-occur within the older acutely admitted patient. Indeed, 12.1% and 7.7% of patients displayed non-sarcopenic frailty or non-frail sarcopenia, respectively, highlighting the necessity of separate assessments of frailty and sarcopenia to ensure the accurate characterization of the health status of older patients.

*Funding:* The work is supported by funding from the Novo Nordisk Foundation; grant number NNF18OC0052826. Open access funding provided by Copenhagen University.

*Conflict of interest:* All authors declare no conflict of interest.

*Compliance with Ethical Standards:* All parts of the trial procedures are performed in accordance with the Declaration of Helsinki.

*Ethical approval:* The study protocol was approved by the local ethics committee of Copenhagen and Frederiksberg (ID: H-19039214).
